# Prevalence of Internet Gaming Disorder in Medical Students: A Meta-Analysis

**DOI:** 10.3389/fpsyt.2021.760911

**Published:** 2022-01-24

**Authors:** Christine L. L. Chiang, Melvyn W. B. Zhang, Roger C. M. Ho

**Affiliations:** ^1^Yong Loo Lin School of Medicine, National University of Singapore, Singapore, Singapore; ^2^National Addictions Management Service (NAMS), Institute of Mental Health, Singapore, Singapore; ^3^Department of Psychological Medicine, Yong Loo Lin School of Medicine, National University of Singapore, Singapore, Singapore; ^4^Institute of Health Innovation and Technology (iHealthtech), Singapore, Singapore

**Keywords:** internet gaming disorder, medical students, psychiatry, meta-analyses, addiction

## Abstract

**Introduction:**

In the last decade, the technological advances have led to increased usage of the Internet. Internet-based games are now more readily available, and they are also more attractive and engageable for individuals. Previous studies have established university students as vulnerable population with regards to IGD. Despite the unique stressors and demands of the course, there is little information about the pooled prevalence of IGD in medical students.

**Objectives:**

The current meta-analysis aims to establish the pooled prevalence of IGD among medical students in different countries, and factors affecting the pooled prevalence.

**Methods:**

A comprehensive search was conducted from 23 May 2021 to 30 May 2021. The following databases were searched: PubMed, PsychINFO, Cochrane CENTRAL, Google Scholar, Scopus, Web of Science, Embase, Science Direct. The search terminologies included permutations of the keywords for IGD and medical students. All statistical analysis was performed with the Comprehensive Meta-analysis Version 3.0 program based on random-effects model.

**Results:**

Three hundred ninety-six articles were identified from the search of the databases. The final data set consisted of 6 studies with a pooled cohort size of 2,236 medical students. The pooled prevalence of IGD in each country is as follows: Egypt had the highest prevalence rate of 10.9% (95% CI: 7.3–16.1%), followed by Saudi Arabia (8.8, 95% CI: 5.7–13.2%), Indonesia (6.1, 95% CI: 0.7–37.5%) and India (3.8, 95% CI: 2.7–5.5%) (*p* < 0.005).

**Conclusions:**

In conclusion, this meta-analysis reports that the pooled prevalence of IGD among medical students from different countries is 6.2%, which is around twice as high than that of the general population.

## Introduction

Over the last decade, advances in gaming technology, increased availability and affordability of high-speed internet have made internet gaming more engaging and attractive than ever (1, 2). There has been a growing recognition of internet gaming disorder (IGD) as a psychiatric condition that is associated with significant impairment and distress (3). A review by Mihara et al. reported that the global prevalence of IGD ranged from 0.7 to 27.5% (4), while another review by Stevens et al. reported the global prevalence of IGD to be 3.05% (5). The variation in prevalence of IGD has been attributed to the lack of consensus in the diagnosis of IGD, with there being heterogeneity in the choice of instruments, the diagnostic and intervention processes for IGD (4–6). However, the Diagnostic and Statistical Manual of Mental Disorders Fifth edition (DSM-5) and International Classification of Diseases 11 (ICD-11) do provide guidelines to identify individuals affected by IGD. The DSM-5 has proposed IGD as a condition that warrants further research evaluation. The DSM-5 states that an individual is likely to have the disease if they have at least 5 out of 9 diagnostic criteria: preoccupation, psychological withdrawal, tolerance, failure to cut down or stop, loss of interest in previously enjoyable pursuits, constant gaming despite ramifications, lying about the extent of use, and gaming to cope with negative affect, or that gaming has led to significant psychosocial impairments. More recently, the ICD-11 formally recognizes Internet gaming as a disorder. Based on the ICD-11, gaming disorder could be classified into either online or offline gaming disorder, and individuals are diagnosed with the disorder if they cannot control the use of games, or have been prioritizing gaming over other activities, or have had exacerbated gaming behavior despite negative life consequences. Prior to these characterizations and the inclusion of gaming disorder in the respective diagnostic manuals, researchers have investigated into the issues of problematic Internet use (PIU) In fact, gaming-related disorder might be one of the subtypes of PIU, as highlighted by Fineberg et al. (7). Other issues related to PIU include that of compulsive sexual behaviors, purchasing items on the Internet, gambling or the excessive use of social media (7).

Whilst prior studies have examined the overall prevalence of IGD in the general studies, other studies have highlighted the importance of studying IGD in university students (8, 9). Today, the Internet is freely available in university campuses for the promotion of academic activities. However, this has made internet gaming an easily accessible and popular recreational activity, making university students vulnerable to IGD (9). A study of 7,022 1st-year college students in Mexico found a IGD prevalence rate of 5.2% (9). In China, a study of 922 university freshmen in Hunan province found an IGD prevalence rate of 5.5% (10). One study of 221 Italian university students found a prevalence rate as high as 14.9% of IGD (11). Medical students may be more vulnerable to developing an addiction to games, due to the additional and unique challenges they face as compared to other university students. A study conducted in Ain-Shams University, Egypt found that the prevalence of IGD was 10.9% among students in the medical faculty, as compared to 9.3% in the overall university student body (12). Medical students often face more academic pressure and a longer duration of study compared to other university students (13). The increased emotional and academic demands of medical school can have a negative effect on medical students, and can precipitate psychopathologies such as depression, anxiety, and burnout (14, 15). This is reflected in the higher rates of anxiety (14) and addictive behaviors in medical students compared to the general population (16). A previous meta-analysis found a high pooled prevalence of internet addiction (30.1%) among medical students, approximately five times that of the general population (16). With many medical students turning to the internet as a potential coping strategy, potential effects of IGD and its co-morbidities on medical student's academic and professional performance should be considered. Pathological use of video games may have negative consequences for those affected such as relationship conflicts, sleep problems or occupational functioning (17, 18). Additionally, IGD typically does not present in isolation. Previous research has reported an association between IGD and anxiety, depression, attention deficit hyperactivity disorder (ADHD) symptoms as well as social phobia/anxiety and obsessive-compulsive symptoms (5, 19, 20). IGD and its co-morbidities may also have repercussions on patient care in the long run as psychological distress in medical students is associated with cynicism, decreased empathy and an unwillingness to care for the chronically ill (21).

As discussed previously, previous studies have established university students as vulnerable population with regards to IGD. Despite the unique stressors and demands of the course, there is little information about the pooled prevalence of IGD in medical students. This may hold important implications for educators who should be well-equipped to address IGD in students. This may also have important clinical implications due to the negative effects of IGD on student's professional and academic performance. It is for these reasons that we set out to do this meta-analysis.

Meta-analysis is a statistical procedure used for combining the results of a number of studies in order to estimate a pooled effect size. Previous meta-analysis studied the pooled prevalence of depression (22), anxiety (15) and internet addiction (16) among medical students. However, the pooled prevalence of internet gaming disorder specifically, remains unknown. Hence, the current meta-analysis aims to establish the pooled prevalence of IGD among medical students in different countries. We also aim to identify factors that would affect the pooled prevalence of IGD within this population.

## Methodology

### Search Strategy

A comprehensive search was conducted from 23 May 2021 to 30 May 2021. The following databases were searched since inception of database to May 2021: PubMed, PsychINFO, Cochrane CENTRAL, Google Scholar, Scopus, Web of Science, Embase, Science Direct. The search terminologies included permutations of the keywords for IGD (e.g., Internet gaming disorder, problematic gaming, excessive gaming) and medical students. Further papers were included via a snowballing approach - via the citation references of primary articles.

### Inclusion and Exclusion Criteria

The inclusion criteria for this meta-analysis were as follows: (a) medical students (undergraduate or postgraduate) formed the main cohort of the study population, (b) the severity of IGD was measured by a relevant questionnaire, and (c) prevalence of IGD was the primary outcome of the study population. The exclusion criteria were as follows: (a) a study without an abstract written in English and (b) insufficient information to compute the pooled prevalence of IGD.

### Selection of Articles

Articles were de-identified [author(s), year of publication, and journal name] before data extraction. Selection of the relevant publications was conducted by two authors, namely that of CLLC and MWBZ. In the first phase, articles were screened based on their titles and abstracts. The shortlisted articles were then evaluated against the inclusion and exclusion criteria. Any disagreement was resolved by discussion between the authors, and a consultation with the senior author. This selection procedure was conducted in accordance to preferred reporting items for systematic reviews and meta-analyses (PRISMA) guidelines (23).

### Data Extraction

The following information was extracted from each of the selected articles and recorded on a standardized electronic data collation form: (a) publication details (names of the authors and year of publication), (b) the number of medical students diagnosed with IGD, (c) the total sample size for each study, (d) the mean age of participants, (e) the proportion of male and female participants, and (f) details of the assessment questionnaire used to assess IGD.

### Statistical Analysis

All statistical analysis was performed with the Comprehensive Meta-analysis Version 3.0 program based on random-effects model and methods established in previous studies (16, 24). In the analysis, random-effect modeling was utilized in view of the heterogenous study designs and the sampled population. One of the underlying assumptions of the random-effect model was that there were varying effect sizes between each of the studies. Heterogeneity between the studies was measured using the *I*^2^ statistic, which describes the percentage of variability among effect estimates beyond that expected by chance. If the value is below 25%, it implies there being low heterogeneity. A value of 50% implies moderate, and a value of 75% high levels of heterogeneity.

Meta-regression analysis was performed in order to identify potential factors (both continuous and categorical) that might have contributed to the overall heterogeneity of the pooled effect size. In the meta-regression analysis, the regression coefficients and the associated *z*-values and *p*-values were reported. Subgroup analysis was undertaken to investigate the effects of categorical variables on the pooled prevalence of IGD. We compared the prevalence of IGD between subgroups based on the country and region of the study. To determine the presence of publication bias, Egger's regression test was conducted. If significant publication bias was present, the classic fail-safe test was performed to determine the number of missing studies that are required such that the *p*-value of the publication bias among the observed studies would be >0.05 (22).

To identify factors that may contribute to the heterogeneity and predict the effective size, meta-regression was performed (25).

## Results

**Figure 1:**
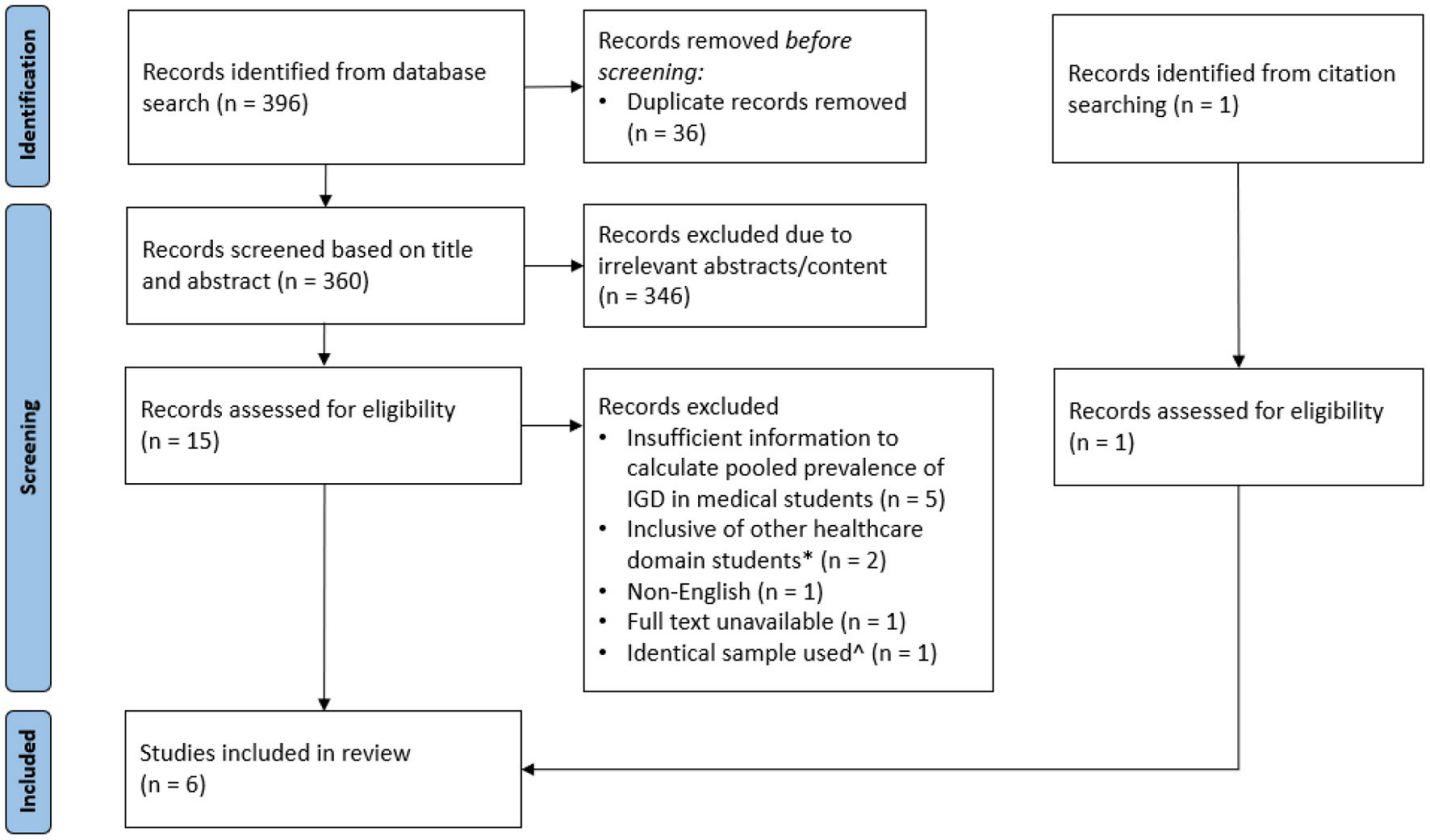
PRISMA flow chart showing selection of studies.

Three hundred ninety-six articles were identified from the search of the databases as well as citation searching and 36 duplicate records were removed before screening. Three hundred sixty unique articles were screened based on title and abstract. In total, 16 full texts were reviewed, with 1 article retrieved through citation references from a primary article. From review of the full text articles, 5 were excluded due to insufficient information to calculated pooled prevalence of IGD. Two studies were excluded because they included subjects who were studying other healthcare domains (e.g., dentistry) along with medicine. One study was excluded because it did not provide access to the full text. One study was excluded as it was not in English. One study was excluded since it used an identical sample to another study. The final data set consisted of 6 studies with a pooled cohort size of 2,236 medical students. Figure 1 shows the selection of the articles. The characteristics of studies included are described in Table 1.

**Table 1:** Baseline characteristics of studies included.

**References**	**Study location**	**Study design**	**Prevalence of internet gaming disorder (%)**	**Number of students addicted (n)**	**Total sample size (n)**	**Proportion of male students (%)**	**Proportion of female students (%)**	**Mean age (years)**	**Characteristics of medical students**	**IGD assessment questionnaire**
Al Asqah et al. (26)	Saudi Arabia	Cross-sectional	8.77%	20	228	64.90	35.10	21.15	Undergraduate	Internet Gaming Disorder 9-Item Short Scale (26)
Balhara et al. (27)	India	Cross-sectional	3.59%	11	306	46.73	53.27	22.73	Undergraduate and Postgraduate	Internet Gaming Disorder Scale-Short Form (27)
Basu et al. (28)	India	Cross-sectional	4.01%	17	424	62.30	37.70	19.83	Undergraduate	Internet Gaming Addiction Scale (29)
Kurnianingsih et al. (30)	Indonesia	Cross-sectional	16.67%	73	438	37.13	62.87	20.2	Undergraduate	Personal Internet Gaming Disorder Evaluation-9 (30)
Siste et al. (31)	Indonesia	Cross-sectional	2.03%	13	639	64.20	35.80	19.9	Undergraduate	10-item Internet Gaming Disorder Test (31)
Elnahas et al. (12)	Egypt	Cross-sectional	10.95%	22	201	NIL	NIL	NIL	Undergraduate	Internet Gaming Disorder 9-Item Short Scale (26)

**Figure 2:**
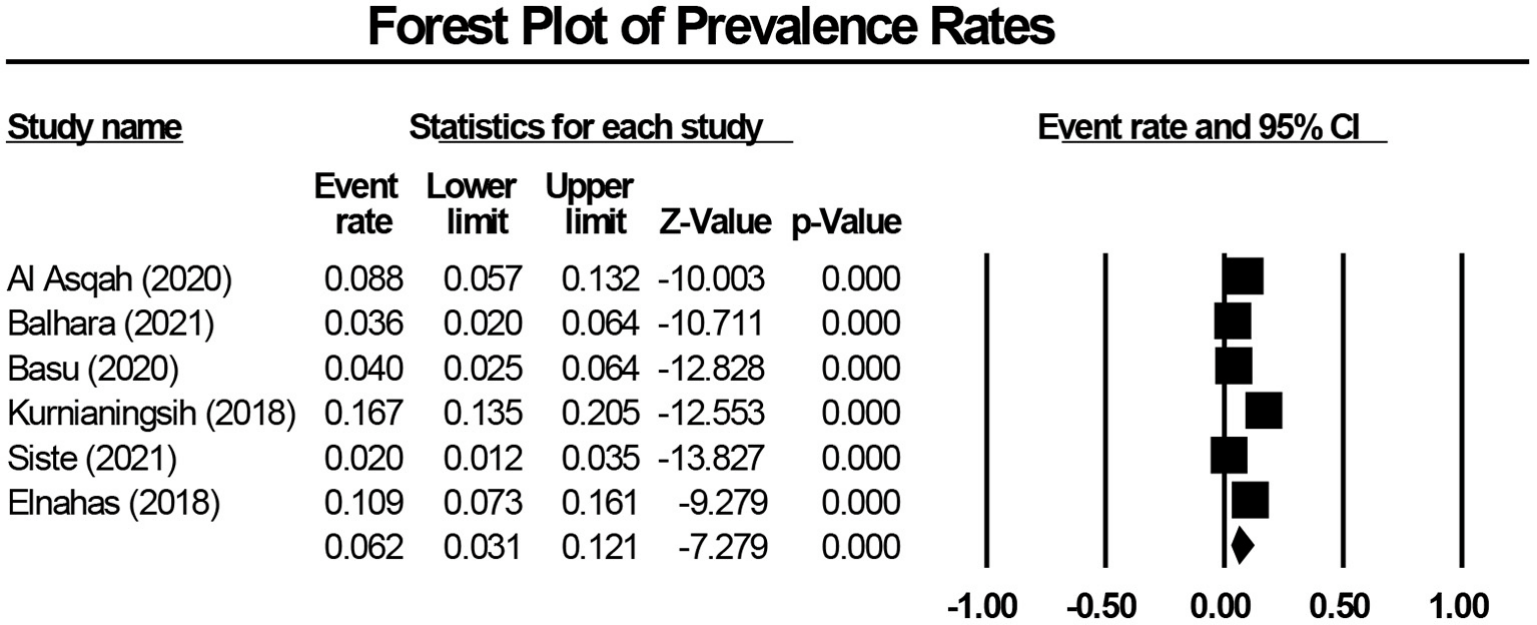
Forest plot of pooled prevalence of internet gaming disorder among medical students.

The pooled prevalence of IGD among 2,236 medical students using the random-effects model was 6.2% (95% CI: 3.1–12.1%, *Q*-value = 84.306, df = 5, *p* < 0.001, tau^2^ = 0.774, *I*^2^ = 94.069). This meta-analysis demonstrates significant heterogeneity across studied (*p* < 0.0001). Figure 2 shows the forest plot generated for the pooled prevalence of IGD among medical students. In the meta-regression analyses (maximum likelihood) (Table 2), mean age (β = −0.1366, *Z* = −6.31, *p* < 0.001) and proportion of males (β = −5.0989, *Z* = −10.35, *p* < 0.001) were non-significant moderators of the overall pooled prevalence of IGD.

**Table 2:** Meta-regression analysis on the sources of heterogeneity.

**Predictor**	**No. studies**	**Univariate coefficient**	***Z*-value**	***P*-value**	**Estimated tau^**2**^**	***R* ^2^**
Mean age of participants	5	−0.0357	−0.11	0.9159	0.5965	−0.6243
Proportion of females, %	5	4.2368	1.69	0.0917	0.3521	−0.8561

Based on the subgroup analysis (Table 3), the pooled prevalence of IGD in each country is as follows: Egypt had the highest prevalence rate of 10.9% (95% CI: 7.3–16.1%), followed by Saudi Arabia (8.8, 95% CI: 5.7–13.2%), Indonesia (6.1, 95% CI: 0.7–37.5%) and India (3.8, 95% CI: 2.7–5.5%) (*p* < 0.005).

**Table 3:** Subgroup analysis on sources of heterogeneity.

**Subgroup**	**No. of studies**	**Pooled prevalence (%)**	**95% CI**	***p*-value in between group comparisons**
Egypt	1	10.9	7.3–16.1	0.00^*^
Saudi Arabia	1	8.8	5.7–13.2	
Indonesia	2	6.1	0.7–37.5	
India	2	3.8	2.7–5.5	

* *p < 0.05 is considered significant*.

Using Egger's regression test, publication bias was evident in the meta-analysis of all studies (intercept = −10.80, 95% CI: −17.98 – −3.62, *t* = 4.18, df = 4, *p* = 0.007). Based on the classic fail-safe test, 1,241 missing studies are required for every observed study in order to nullify the result.

## Discussion

The main finding from our meta-analysis is the pooled prevalence rate of IGD among 2,236 medical students of 6.2%. This was noted to be double that of global prevalence of IGD of 3.05% reported in Steven's meta-analysis (5). Another review found the prevalence of IGD in adolescents to be between 1.3 and 19.9% (4). As discussed above, this serves to highlight the potential vulnerability of medical students to developing IGD. However, this study also highlights the methodological differences used across studies, with 5 different assessment tools used in the 6 studies included. Stevens found in their meta-analysis that choice of screening tool accounted for 77.97% of the variance across prevalence estimates (5).

Meta-regression analysis found that age is not a significant moderator of prevalence rate of IGD in medical students and does not account for the heterogeneity in the pooled prevalence of IGD in medical students. However, Kurnianingsih et al. (30) and Al Asqah et al. (26) found that the prevalence of IGD was higher in the earlier years in medical school. Al Asqah et al. (26) found that the majority (65%) of the students found to have IGD was from the 1st and 2nd years while Kurnianingsih et al. (30) found that Year 1 medical students had the highest prevalence of IGD at 32%. The 1st year of entering university education is a crucial period, where students have to begin to handle the demands for independent functioning and the higher academic demands that often accompany the university transition (32). First year medical students tend to have higher stress level than students of 2nd, 3rd, and 4th year (33). With stress symptoms being a risk factor for developing IGD (34), the stress of the transition period could contribute to higher rates of IGD in younger medical students.

The meta-regression analysis which we have undertaken revealed that male gender was not a significant moderator, and it did not account for the high heterogeneity in the pooled prevalence of IGD we reported. This is not congruent with the findings of prior studies, in that they reported the rates of IGD to be higher amongst males, in comparison to females (5). Prior studies explained the discrepancy in the rates by highlighting that males tend to be prone toward gaming (5), whereas females tend to be prone toward the use of social networking sites (4, 35, 36). One of the other contributing factors is that the gaming industry has been focused mainly on targeting males, and this could have increased the vulnerability of males in developing IGD (37). In spite of this, in our results, we found an exception to this trend. Few empirical studies have specifically studied female gamers, with almost all research on IGD focusing on male gamers (38). However, one study found that there were gender differences in how IGD scores were predicted by several variables. Female gamer's IGD predictors included higher time spent online and higher scores in competition as gaming motives. In contrast, male gamer's IGD predictors included higher scores in coping and higher depressive symptoms compared to females (39). More can be done to research potential links between IGD and female medical students, such as the differing motives and predictors that female medical students may have. Several factors with regards to the studies included could possibly explain this as well. Kurnianingsih et al. (30) reported the lowest proportion of males (37.13%) but the highest prevalence of students with IGD (16.67%). This study had a large sample size of 438, which was around 19.6% of the total pooled sample size used. This could have influenced the results.

From the subgroup analysis, the pooled prevalence of IGD in the Middle Eastern countries was higher than in India and Indonesia. The Middle East and North Africa (MENA) region is a fast-growing gaming market with its growing community of active gamers and high internet penetration. However, it should be considered that both Middle Eastern studies used Lemmen's IGD 9-item Short Scale (40) which was found to have report higher prevalence estimates in a previous meta-analysis (5). This could be due to the dichotomous nature of the IGD 9-Item Short Scale. The scale consists of 9 yes-or-no questions based on the DSM-5 criteria for IGD, with a point awarded for each question answered with “yes.” A score of 5 or higher indicates a disordered gamer.

This meta-analysis has several strengths. Firstly, to our knowledge, this is the first meta-analysis that examined the prevalence of IGD among medical students in different countries. Secondly, our search strategy has included a wide range of databases. Despite these inherent strengths, one of the major limitations of this review was the number of studies that could be identified. We were limited to the identification of only 6 studies, and this small nature might be since medical students are a rather unique population of interest, and this being a niche area for scientific investigation. In addition, the studies used are based on 4 different countries (2 from India, 2 from Indonesia, 1 from Saudi Arabia, 1 from Egypt). We could not locate studies conducted Europe, North America, East Asia, and Australia during the search. This limitation is noteworthy because it limits the generalizability of the results. Further studies focusing on the prevalence of IGD among medical students are required from these continents. Secondly, the studies included in this meta-analysis were all cross-sectional. Hence, this meta-analysis is not able to demonstrate causality or temporal association between IGD and medical studies. Thirdly, we could not perform subgroup analysis to compare the prevalence of IGD between undergraduate and postgraduate medical students due to the nature of the reviews available. The study by Balhara et al. (27) included in the review had a mixed sample of both undergraduate and postgraduate medical students.

In terms of research implications, this study reinforces the need for a standardized, validated questionnaire to ascertain prevalence of IGD. Estimates of prevalence of IGD continue to be associated with significant variability (5). While many assessment tools may appear to be quite similar, they have been found to demonstrate varying psychometric properties (41). Research regarding IGD is a rapidly evolving field and findings may change with new studies being conducted. More studies involving medical students could be conducted in areas with high internet prevalence rates that were not included in this review. Notably, East Asian, North American, European and Australian cohorts. With the subgroup analysis finding differences in prevalence of IGD across different regions significant, further studies could be done to compare cohorts in such regions. Moreover, with the onset of COVID-19, there is likely a change in the usage patterns amongst individuals, and greater reliance on the Internet for academic purposes. In a recent narrative review by Gjoneska et al. (7), the authors have also highlighted several research priorities upon consideration of the impact of COVID-9 on such problematic behaviors. Some of the research priorities that might be relevant to this current study include the need to also examine the psychological well-being of medical students who are gaming excessively.

This study has clinical implications for medical school administrators and teachers. With a greater understanding of the signs and symptoms of IGD, prompt recognition could allow for early referral and intervention by mental health professionals. This would allow for further deterioration of the student's IGD and potential negative effects (42). It is also of importance to screen for other psychiatric comorbidity including sleep deprivation. With IGD having links to poorer sleep quality (43) and increased psychological distress (20), this could lead to impairments in medical student's ability to perform academically and professionally. With sleep deprivation's detrimental effects on medical student's attention performance (44) and decision making (45), this is of concern for medical students and patient safety as well. By teaching about IGD, medical educators can raise awareness of the condition amongst medical students. They can also offer cognitive behavioral therapy as a first-line therapy, to improve IGD symptoms and comorbid depression (42).

## Conclusions

In conclusion, this meta-analysis reports that the pooled prevalence of IA among medical students from different countries is 6.2%, which is around twice as high than that of the general population. Age and gender cannot explain high heterogeneity in prevalence, but the region in which the study occurs is a potential source of heterogeneity. Given the high prevalence of IGD, medical teachers and medical school administrators should identify medical students suffering from IGD and refer them for intervention. The medical school psychiatric curriculum should cover IGD and increase the awareness of IGD among medical students.

## Author Contributions

CC, MZ, and RH jointly conceptualized the study. CC and MZ reviewed the literature and obtained the primary data. CC performed the meta-analysis, under the guidance of MZ. MZ verified the data-analysis. CC wrote the first draft of the manuscript, which MZ and RH provided critical inputs to. All authors read and approved of the manuscript prior to submission.

## Funding

MZ was supported by a grant under the Singapore Ministry of Health's National Medical Research Council (Grant No. NMRC/Fellowship/0048/2017) for PhD training. The funding source was not involved in any part of this project. This study was funded by NUS Department of Psychological Medicine (R-177-000-100-001/R-177-000-003-001) and NUS iHealthtech Other Operating Expenses (R-722-000-004-731).

## Conflict of Interest

The authors declare that the research was conducted in the absence of any commercial or financial relationships that could be construed as a potential conflict of interest.

## Publisher's Note

All claims expressed in this article are solely those of the authors and do not necessarily represent those of their affiliated organizations, or those of the publisher, the editors and the reviewers. Any product that may be evaluated in this article, or claim that may be made by its manufacturer, is not guaranteed or endorsed by the publisher.
